# Supplementary material for: Self-Template Synthesis of Nitrogen-Doped Hollow Carbon Nanospheres with Rational Mesoporosity for Efficient Supercapacitors
Source: Materials (Basel). 2021 Jun 29;14(13):3619. doi: 10.3390/ma14133619 (PMC8269615; doi:10.3390/ma14133619)
Supplement: Supplementary file 1 [file materials-14-03619-s001.zip › materials-1235337-supplementary.pdf]

materials

Supplementary Materials

Self-Template Synthesis of Nitrogen-Doped Hollow Carbon Nanospheres with Rational Mesoporosity for Efficient Supercapacitors

Xiang Zhao [1,2], Mu Zhang [1,2,*], Wei Pan [1,2], Rui Yang [1,2] and Xudong Sun [1,2,*]

[1] Key Laboratory for Anisotropy and Texture of Materials (Ministry of Education), Northeastern University, Shenyang 110819, China; 1310142@stu.neu.edu.cn (X.Z.); 1510139@stu.neu.edu.cn (W.P.); yangrui@stumail.neu.edu.cn (R.Y.)
[2] Lab of Advanced Ceramics, Foshan Graduate School of Northeastern University, Foshan 528311, China
* Correspondence: zhangm@mail.neu.edu.cn (M.Z.); xdsun@mail.neu.edu.cn (X.S.)

Citation: Zhao, X.; Zhang, M.; Pan, W.; Yang, R.; Sun, X. Self-Template Synthesis of Nitrogen-Doped Hollow Carbon Nanospheres with Rational Mesoporosity for Efficient Supercapacitors. *Materials* **2021**, *14*, 3619. https://doi.org/10.3390/ma14133619

Publisher's Note: MDPI stays neutral with regard to jurisdictional claims in published maps and institutional affiliations.

Copyright: © 2021 by the authors. Licensee MDPI, Basel, Switzerland. This article is an open access article distributed under the terms and conditions of the Creative Commons Attribution (CC BY) license (http://creativecommons.org/licenses/by/4.0/).

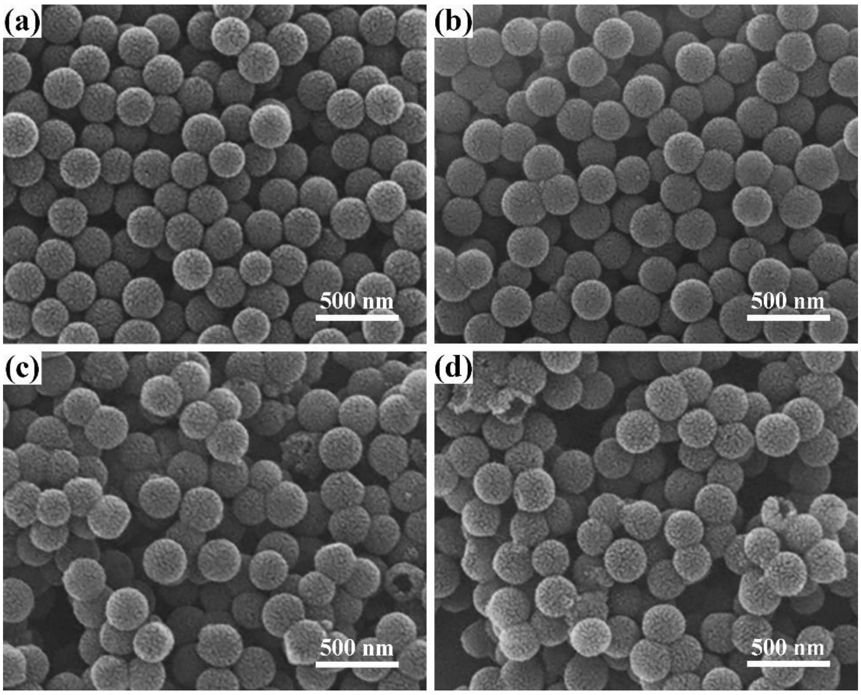

Figure S1. SEM images of the resulting NHRFSs, which were further labeled as NHRFS-*m*, where "NHRFS" refers to nitrogen-doped hollow resorcinol-formaldehyde nanospheres, and symbol "*m*" represents the AFC dosage used (in mg): (**a**) NHRFS-0; (**b**) NHRFS-10; (**c**) NHRFS-30; and (**d**) NHRFS-50, respectively.

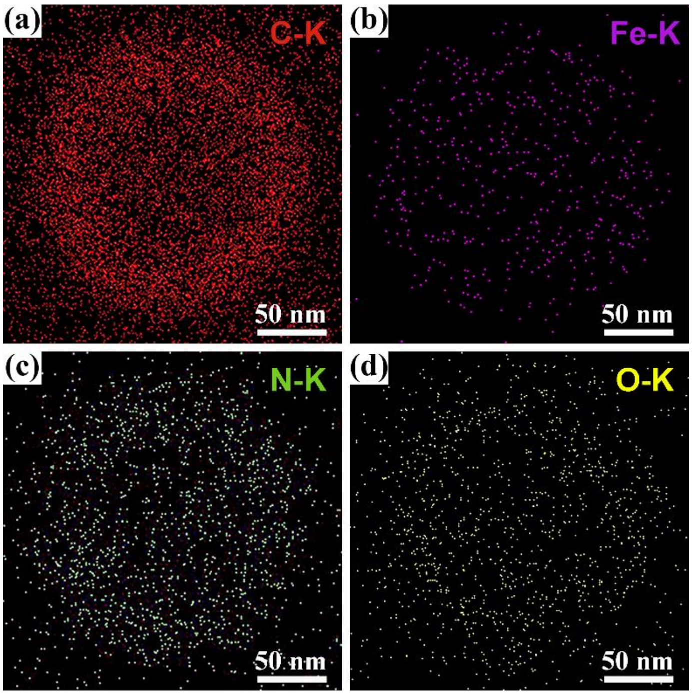

Figure S2. EDS elemental mapping images of NHCS-30 (the selected particle is shown in Figure 2o).

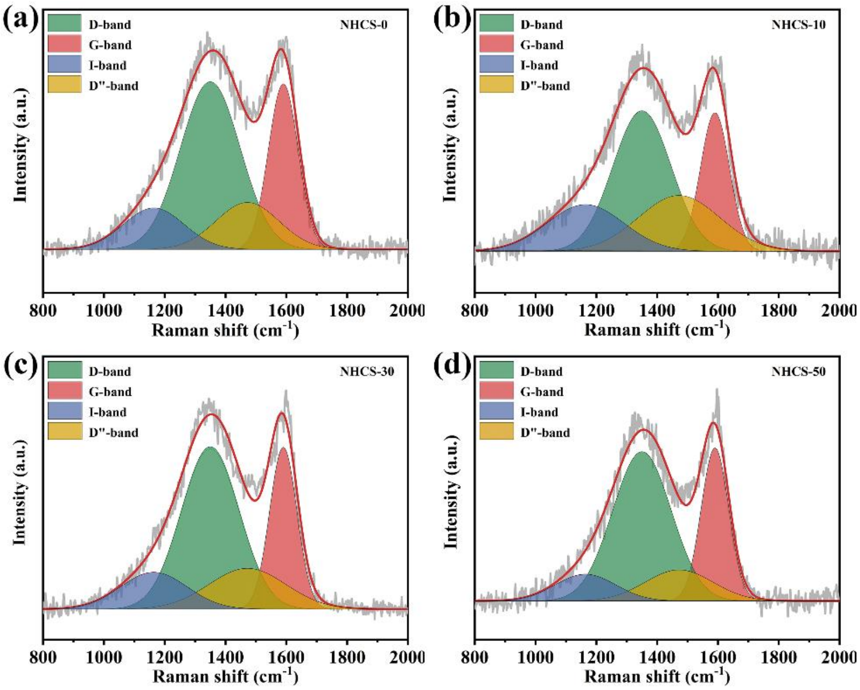

Figure S3. Raman-peak-differentation-imitating-analysis for NHCS-*m*.

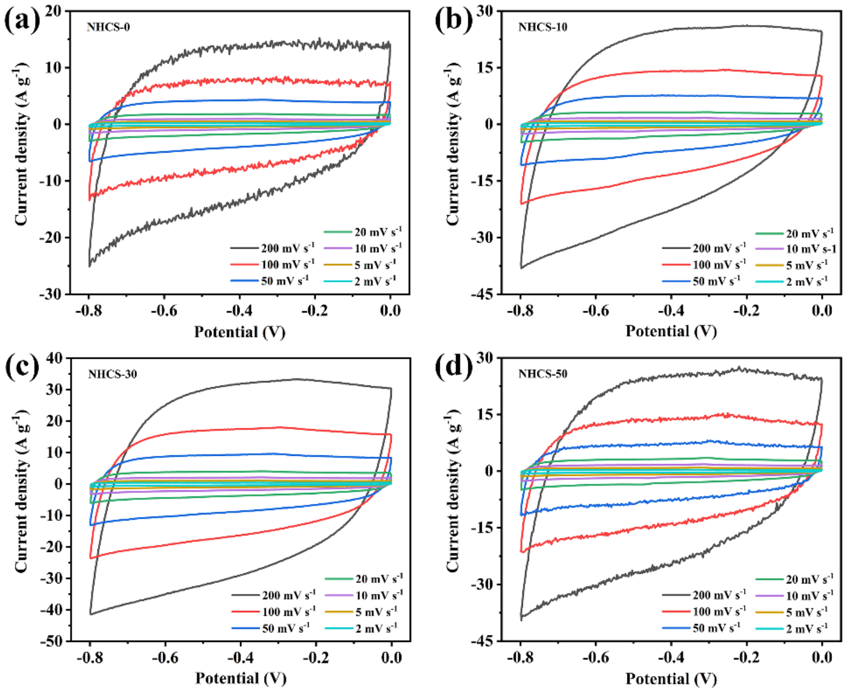

Figure S4. CV curves at various scan rates (from 2 to 200 mV·s^{-1}) for (**a**) NHCS-0, (**b**) NHCS-10, (**c**) NHCS-30, and (**d**) NHCS-50.

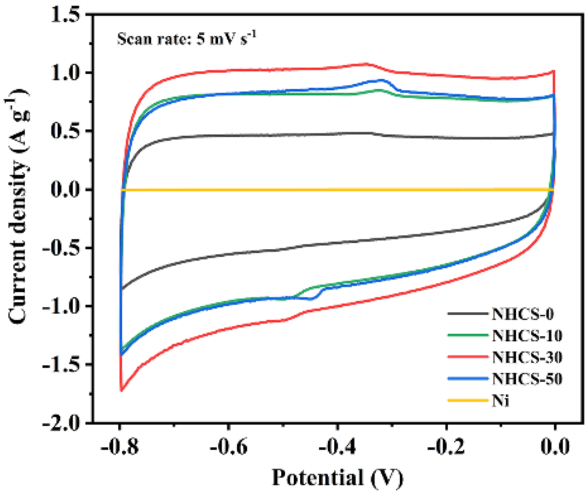

Figure S5. CV curves of the NHCS-m electrodes at a fixed scan rate 5 mV·s^{-1}.

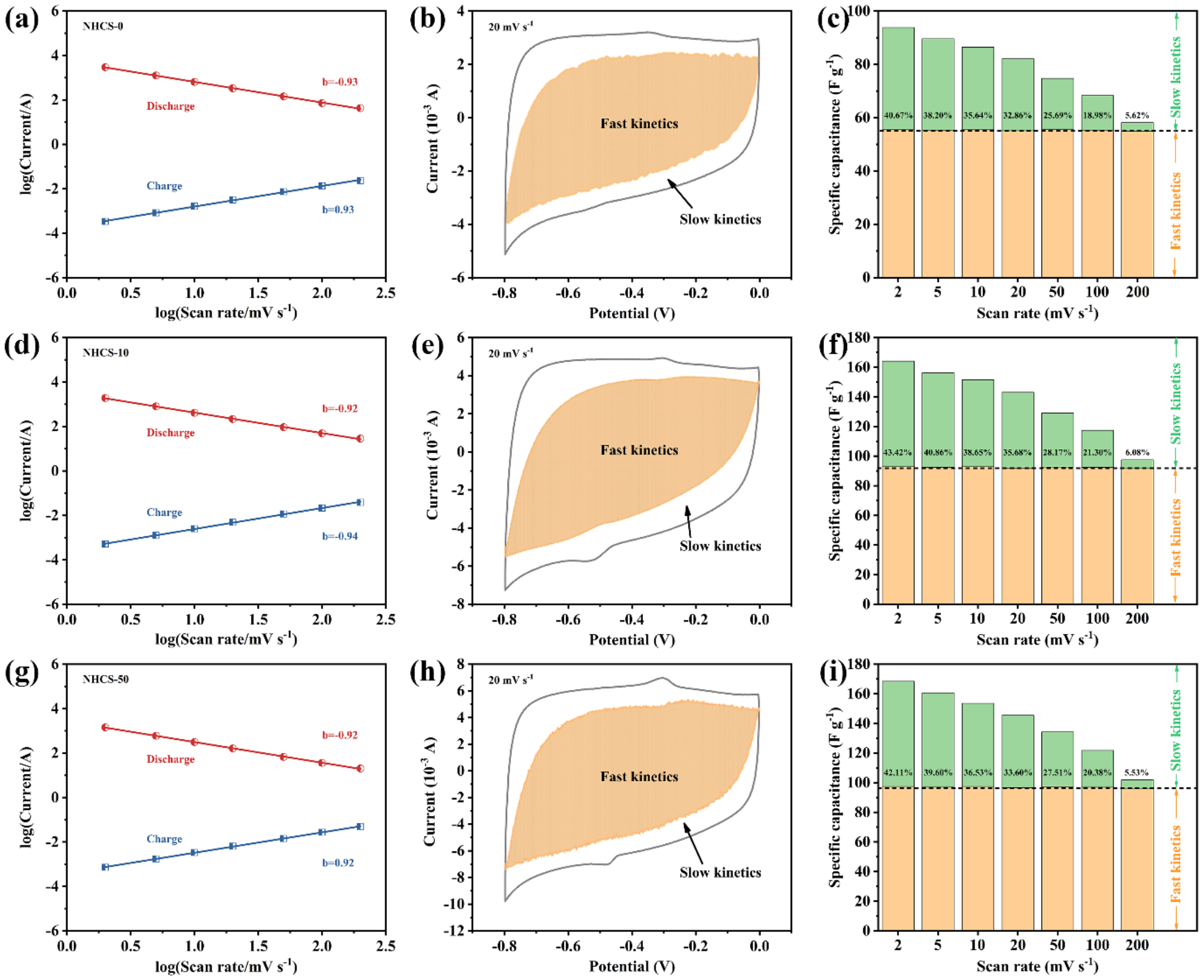

Figure S6. (**a**,**d**,**g**) Relationship between log(current) and log(scan rate) in the charge/discharge process for NHCS-0, NHCS-10, and NHCS-50 respectively; (**b**,**e**,**h**) Decoupling of the capacity contributed by the fast kinetic process (shadow) for NHCS-0, NHCS-10, and NHCS-50 respectively; (**c**,**f**,**i**) Histograms of capacitive contribution ratio at various scan rates for NHCS-0, NHCS-10, and NHCS-50 respectively.

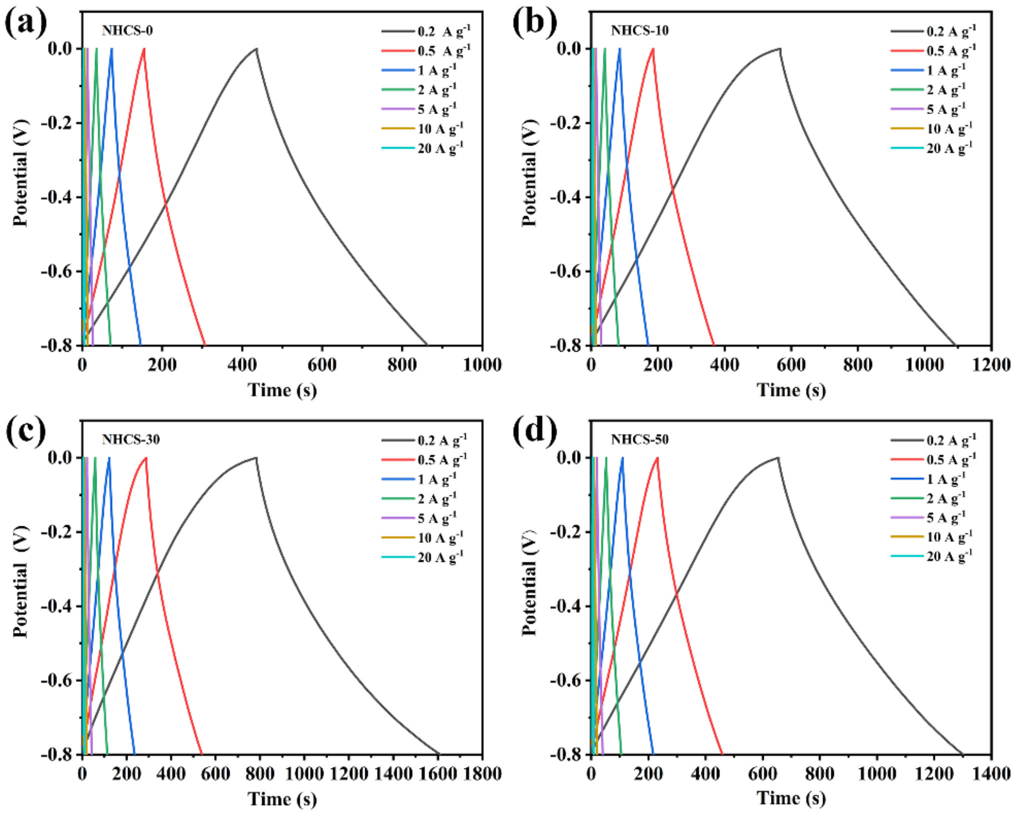

Figure S7. GCD curves at various current densities (from 0.2 to 20 A g^{-1}) for (**a**) NHCS-0, (**b**) NHCS-10, (**c**) NHCS-30, and (**d**) NHCS-50.
